# The impact of decision tools during oncological consultation with lung cancer patients: A systematic review within the I3LUNG project

**DOI:** 10.1002/cam4.7159

**Published:** 2024-05-14

**Authors:** Valeria Sebri, Chiara Marzorati, Patrizia Dorangricchia, Dario Monzani, Roberto Grasso, Arsela Prelaj, Leonardo Provenzano, Laura Mazzeo, Andra Diana Dumitrascu, Jana Sonnek, Marlen Szewczyk, Iris Watermann, Francesco Trovò, Nina Dollis, Evangelos Sarris, Marina Chiara Garassino, Christine M. Bestvina, Alessandra Pedrocchi, Emilia Ambrosini, Sokol Kosta, Enriqueta Felip, Mireia Soleda, Aina Arbusà Roca, Jose Rodríguez‐Morató, Alessandro Nuara, Yonah Lourie, Melissa Fernandez‐Pinto, Alfonso Aguaron, Gabriella Pravettoni

**Affiliations:** ^1^ Applied Research Division for Cognitive and Psychological Science IEO, European Institute of Oncology IRCCS Milan Italy; ^2^ Laboratory of Behavioral Observation and Research on Human Development, Department of Psychology, Educational Science and Human Movement University of Palermo Palermo Italy; ^3^ Department of Oncology and Hemato‐Oncology University of Milan Milan Italy; ^4^ Thoracic Oncology Unit, Medical Oncology Department 1 Fondazione IRCCS Istituto Nazionale Tumori Milan Italy; ^5^ Department of Electronics, Information, and Bioengineering Politecnico di Milano Milan Italy; ^6^ Medical Oncology Department Fondazione IRCCS Istituto Nazionale dei Tumori di Milano Milan Italy; ^7^ Lungen Clinic Grosshansdorf, Airway Research Center North German Center for Lung Research Grosshansdorf Germany; ^8^ Politecnico di Milano Milan Italy; ^9^ 4th Oncology Dept Metropolitan Hospital; ^10^ Knapp Center for Biomedical Discovery University of Chicago Medicine & Biological Sciences Chicago Illinois USA; ^11^ Department of Electronics, Information and Bioengineering Neuroengineering and Medical Robotics Laboratory NearLab Milan Italy; ^12^ Department of Electronic Systems Aalborg University Copenhagen Denmark; ^13^ Vall d'Hebron University Hospital Barcelona Spain; ^14^ Vall d'Hebron Institute of Oncology Barcelona Spain; ^15^ Medica Scientia Innovation Research (MEDSIR) Barcelona Spain; ^16^ Shaare Zedek Medical Center Jerusalem Israel; ^17^ Lung Cancer Europe (LuCE) Bern Switzerland

**Keywords:** anxiety, decision‐making, non‐small cell lung cancer, patient knowledge, treatment consultation

## Abstract

**Introduction:**

To date, lung cancer is one of the most lethal diagnoses worldwide. A variety of lung cancer treatments and modalities are available, which are generally presented during the patient and doctor consultation. The implementation of decision tools to facilitate patient's decision‐making and the management of their healthcare process during medical consultation is fundamental. Studies have demonstrated that decision tools are helpful to promote health management and decision‐making of lung cancer patients during consultations. The main aim of the present work within the I3LUNG project is to systematically review the implementation of decision tools to facilitate medical consultation about oncological treatments for lung cancer patients.

**Methods:**

In the present study, we conducted a systematic review following the PRISMA guidelines. We used an electronic computer‐based search involving three databases, as follows: Embase, PubMed, and Scopus. 10 articles met the inclusion criteria and were included. They explicitly refer to decision tools in the oncological context, with lung cancer patients.

**Results:**

The discussion highlights the most encouraging results about the positive role of decision aids during medical consultations about oncological treatments, especially regarding anxiety, decision‐making, and patient knowledge. However, no one main decision aid tool emerged as essential. Opting for a more recent timeframe to select eligible articles might shed light on the current array of decision aid tools available.

**Conclusion:**

Future review efforts could utilize alternative search strategies to explore other lung cancer‐specific outcomes during medical consultations for treatment decisions and the implementation of decision aid tools. Engaging with experts in the fields of oncology, patient decision‐making, or health communication could provide valuable insights and recommendations for relevant literature or research directions that may not be readily accessible through traditional search methods. The development of guidelines for future research were provided with the aim to promote decision aids focused on patients' needs.

## 1 INTRODUCTION

Lung cancer is considered the second most common cancer worldwide. Generally, it has a negative prognosis due to a low life expectancy.^1^ Of all lung cancer diagnoses, 80% to 85% are attributed to non‐small cell lung cancer (NSCLC). The stage of disease impacts on choosing the best option of treatment, which can vary between radiation, surgery, and systemic therapies (i.e., immunotherapy, target therapy, and/or chemotherapy).^2^ The great variability of available treatments—often combined may highlight the need for information and clarity about the best treatment option. Lung cancer patients often ask for information about their symptoms and the treatment options available. No less important, they want to know the potential side effects in the short and long term. Moreover, in the case of failure to respond to oncological treatments, they may experience high levels of uncertainty and mood disorders.^3^ After receiving a diagnosis, patients need comprehensive information about the disease characteristics, prognosis, and potential follow‐up examinations. Especially during the diagnostic phase, patients seek reassurance regarding the disease's features, prognosis, and upcoming treatment options. Even if their needs may slightly change over the disease trajectory and may vary along different stages, research has demonstrated a desire to receive answers regarding psychosocial and physical illness management.^4^, ^5^ More specifically, a recently published systematic review^5^ on the unmet needs in lung cancer patients highlighted the importance of understanding how to cope with physical symptoms, the demands of treatment, and a need to improve patient well‐being and decision.^3^, ^5^, ^6^

The insufficient information and understanding of side effects were linked to the Quality of Life and psychological well‐being of patients. Lung cancer patients often report significant symptom burden and possess limited knowledge of strategies to manage both short‐term and long‐term side effects. Consequently, this reduce patients' awareness and alters their attitudes toward the disease.^5^ Moreover, compromised physical functioning and unmet needs can result in psychological distress, anxiety, and depression, contributing to mental exhaustion and impacting treatment adherence.^4^ To alleviate this condition and improve patient compliance with treatment, various tools have been developed. These tools aim to heighten patient awareness, and cater to individual preferences and values, thereby fostering empowerment and informed decision.^7^, ^8^ For instance, the implementation of patient decision aids (PDAs) is typically intended to foster a deeper comprehension of disease characteristics and enhance acceptance of clinical outcomes in alignment with psychological needs.^9^ Nonetheless, it could lead to both risks (potential negative outcomes or consequences associated with whatever action or situation is under discussion in the context of the patient's psychological well‐being, —these “risks” could involve heightened anxiety, depression, distress, reduced quality of life, and a negative impact on coping mechanisms) and benefits concerning the patients' psychological well‐being. It is crucial to note that the specific risks will vary depending on the individual patient, their unique circumstances, and the nature of the medical situation under consideration. Additionally, although certain actions or decisions may pose potential risks, there may also be corresponding benefits or positive outcomes to consider.

Over time, PDAs have been increasingly used to help patients to identify the best treatment options, consider their personal values, and enhance awareness of their cancer care encompassing disease characteristics and potential side effects. Consequently, PDAs facilitate the analysis of multifactorial aspects and needs, weighing up each element, and involving patients in the decision‐making process.^10^, ^11^ Recent studies provide scientific evidence regarding the implementation of the PDAs to support decisions in patients with lung cancer.^9^, ^12^ Clark et al.^7^ demonstrated that PDAs increased lung cancer screening knowledge, and enabled patients to rank possible consequences in terms of risks and benefits. Similarly, Manners et al.^13^ demonstrated that implementing of a DA tool, such as an informative pamphlet about lung cancer screening, can enhance the alignment of screening preference with eligibility by reducing decisional conflict.

Existing systematic reviews have already covered the screening phase, with only one systematic investigating the availability and effectiveness of decision aids, conducted by Spronk et al.^12^ However, Spronk et al.^12^ focused on advanced lung cancer patients and limited their study selection to the period between 2006 and 2018. To provide a more comprehensive framework, we aim to systematically review research studies focused on the implementation of decision tools in the lung cancer field. Specifically, we investigated how PDAs can improve patient' awareness and decisions within the I3LUNG project. The European I3LUNG project is funded under the framework of the H2020 call “Ensuring access to innovative, sustainable and high‐quality health care” and is focused on the development and validation of an integrated and easily accessible online platform. By analyzing data from non‐small lung cancer patients using Artificial Intelligence, the current project aims to predict the outcomes of immunotherapy treatments. The goal is to enhance the quality of life and life expectancy for lung cancer patients and develop an ad hoc Individual Patient Decision Aid System (IPPDA). Consequently, this systematic review seeks to offer evidence regarding the effectiveness of PDA tools their related outcomes, including patients' knowledge, emotional well‐being, and shared decision‐making (SDM).

## 2 METHODS

This systematic review was registered with the International Prospective Register for Systematic Reviews. Its ID number is CRD42023393082.

### 2.1 Search strategy

We interrogated the following three databases: EMBASE, SCOPUS, and PUBMED. An online literature search was performed on July 1, 2022. This systematic review has been conducted following the PRISMA guidelines.

### 2.2 Study selection

Records were searched for using: “lung cancer care” and/or “non‐small cell lung cancer” and/or “decision aid” and/or “patient decision aid” and/or “shared decision making” and/or “NSCLC”. Only quantitative research studies were considered for inclusion. Qualitative studies, reviews, editorials, theoretical articles, and study protocols were excluded. We included articles that explicitly refer to PDAs as tools to support decisions in lung cancer patients. In line with the literature, decision aids are defined thus: “A working definition of “decision aid” must recognize that decision aids range in complexity and technology. In essence, a decision aid is a tool for helping the decision aid user solve a problem by presenting the user with some type of embedded information. The tool may be as simple as a formula to be memorized or a paper checklist” (see Wheeler & Murthy, 2011, page 161).^14^

Furthermore, studies with at least one experimental group of lung cancer patients were included. Then, we assessed full text of each article considering the following eligibility criteria:

1. studies that applied a decision aid procedure in the context of a patient–clinician interaction;
2. articles that evaluated the impact of PDAs on patient treatments and psychological implications (e.g., anxiety, coping strategies, and depression);
3. studies that involve lung cancer patients as a patient cohort.

Studies included were all retrieved from peer‐reviewed scientific journals and published in English. As in other reviews,^15^ a priori restriction was applied. Thus, “gray literature” (e.g., other non‐peer‐reviewed sources, doctoral dissertations, and conference abstract) were excluded to improve our review's manageability.^16^ We did not impose other limitations. For example, participants' age, statistical presentation of results, and year of publication were not considered. Outcomes that were analyzed included: patient quality of life, anxiety, decision conflict, patient satisfaction, patient knowledge, and depression.

### 2.3 Coding and selection of studies

The initial search identified 1425 studies. We screened the abstracts of 1128 studies, and we removed duplicates (*n* = 297). For each of the selected studies, three researchers (VS, PD, and CM) independently and in a masked manner performed the initial search and examined all the relevant articles for (1) the basic information (e.g., authors, publication year), (2) the psychological variables assessed by decision aid, (3) the decision aid procedure (patients and clinicians were in oncological consultation); (4) the sample size and sample characteristics (i.e., participants [patients and clinicians]; mean age of participants), and (5) the instruments used in the study and the variables explored. Any discrepancy between researchers was resolved with another author (DM) after a common consensus among another reviewer. Inter‐rater reliability analysis revealed a perfect consensus between researchers. The potential selection bias was assessed through the analysis of Cohen's k. Two raters (JS and YS) independently screened all contributions selected for the full‐text analysis and assessed them for their potential inclusion according to the defined inclusion criteria. Cohen's k for the inter‐rater agreement was 1.00.

**FIGURE 1:**
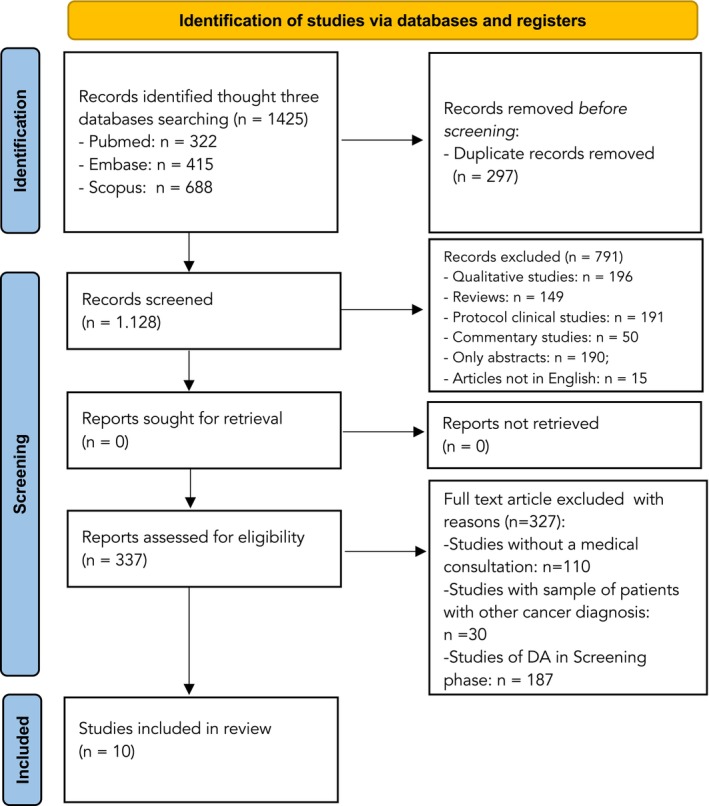
PRISMA diagram flow.

After the first screening phase, the full texts of 337 articles were assessed to identify potential articles that satisfied the aforementioned inclusion criteria. At the end of the screening, 327 contributions were excluded due to not having a decision aid component included. This way, 10 studies were included in the systematic review. The PRISMA guidelines were followed, and all the phases of the review flow are presented in Figure 1.

The authors analyzed dependent variables in the selected studies to categorize them into four outcomes. The first outcome obtained was Anxiety, which appears with a higher frequency compared to the other categories. The second outcome in terms of frequency was related to decision‐making, which was evaluated in terms of decision conflict, satisfaction, preferences, and SDM. The third outcome pertained to Patient Knowledge focusing on the information patients desired to have about their illness and the associated side effects of treatments. The fourth and final outcome defined several variables, such as quality of life, cognitive impairments, depression, frailty, cognitive impairments, information recall, and tool acceptability.

### 2.4 Study retrieval

We included 10 articles in our sample. Table 1 reports the information related to the design and characteristics pertaining to each article included. In order to do this, we considered some thematic categories, as follows: the first author's name and the year of publication, sample, measures and instruments, PDA, and outcomes of interest. Most of the studies under examination showed chemotherapy as the main option of treatment (*N* = 7), while it was not specified for the others (*N* = 3). Notably, only one study^26^ included immunotherapy and targeted therapies among the treatments administered.

**TABLE 1:** Studies included in the present systematic review.

Authors	Study design	Sample	Study aim	Outcomes measures	DAs design	Outcomes of interest
1. Brundage et al. (2001)^17^	Pilot study	27 patients with Locally Advanced NSCLC	To determine whether a decision aid could be implemented in a regional cancer center, examining preliminary findings regarding the criteria for an effective decision aid	1. *Patients knowledge*: *4 open‐ended* ad hoc *questions* ^18^ 2. *Patients preference*: Ad hoc *items* ^19^ 3. *Decisional Conflict*: *Decisional Conflict Scale (DCS)* 20, 21	The decision aid included a structured description of the treatment options and trade‐off exercises designed to clarify the patient's values and survivorship and assess relevant outcomes and advantage threshold	ADs seemed to clarify patients' understanding of relevant information for their treatment decision. At the same time, decisional uncertainty scores decreased, reducing patients' decisional conflict. Moreover, participants who had a clear treatment preference at the beginning of the interview, did not change ideas after using ADs
2. Fiset et al. (2000)^22^	A cross‐sectional survey	20 patients with stage IV of NSCLC	To develop and evaluate a take‐home, self‐administered decision aid, incorporating patient values as an adjunct to counseling	1. *Decisional conflict* *Decisional conflict scale (DCS)* 20, 21 2. *Tool acceptability*: Standardized questions 3. *Patients' knowledge*: ad hoc items^19^	The DAs were a self‐administered, self‐paced booklet, audio‐tape, and worksheet	DAs improved patients' knowledge about treatment options and outcomes, reducing their decisional conflict. Moreover, patients found ADs acceptable and comfortable
3. Hollen et al. (2012)^23^	A prospective study	39 patients with advanced Non‐Small Cell Lung cancer	To assess the feasibility and acceptability of implementing a short, clinic‐based DA aiming at defining an in‐depth patients' clinical profile	1. *Decision‐making quality*: *Decision‐Making Quality Scale (DMQS)* 2. *Decision Conflict*: *Decisional Conflict Scale (DCS)* ^20^, ^21^ 3. *Feasibility of delivery*: *Participant Evaluation Forms* 4. *Anxiety*: *State–Trait Anxiety Inventory (STAI*)^24^	DS intervention was based on “Decision—KEYS for Balancing Choices: Cancer Care” in which patients and their caregivers chose the decision‐role statement that best described their preferred role in decision‐making	Anxiety and decisional conflict, associated with sensation of uncertainty, significantly decreased after receiving DAs. On the contrary, DAs implementation promoted patients' knowledge. In addition, patients reported that DAs were feasible and helped them to make decisions about their treatments. Lastly, decision‐making quality scores were high for both patients and caregivers involved
4. Leighl et al. (2008)^25^	A randomized trial	20 patients with metastatic NSCLC	To develop a decision aid to improve consultations with Metastatic NSCLC patients, facilitating decision‐making	1. *Anxiety*: *the 20‐item State–Trait Anxiety Inventory (STAI Form Y)* ^24^ 2. *Patients' knowledge*: an adapted questionnaire based on *16‐item scale* 3. *Tool acceptability*: an adapted measure based on *16‐item scale*	The DsA was designed as a 25‐page booklet (letter‐sized paper) that included treatment options, toxicity, and survival information illustrated in graphic, numeric, and verbal format. Patients could take home the audio‐recorded booklet	After reviewing the DAs, patient anxiety decreased slightly. Moreover, patients improved their clear understanding of the goals and the awareness of treatment options and their related side effects (e.g., chemotherapy's toxicity). Finally, patients reported an appropriate tool' acceptability.
5. Myers et al. (2021)^26^	A Pilot Study	20 patients with NSCLC	To identify Decision Support Intervention (DSI) that provides information about current treatment alternatives and elicits patients' treatment preference assessing if they can be integrated effectively in routine care	1. *Patients knowledge* *Patient interview and* ad hoc *items* 2. *Decisional conflict*: *Decision Conflict Scale (DCS)* ^20^, ^21^ 3. *Shared Decision‐making*: A *DSI prototype integrated into routine care*	A novel patient‐friendly Decision Support Intervention (DSI) that can be integrated into routine care. DSI provides patients information about current treatment alternatives and elicits their treatment preferences	The deployment of the DSI prototype had a beneficial effect on the perception about treatment, decisional conflict, and patient knowledge. Participants felt more certain about their preferred treatment choices and more aware of their treatment options, decreasing decisional conflict. Moreover, DSI improved Shared Decision‐Making, allowing patients to gain insight into the nature of treatments options
6. Ogawa et al. (2018)^27^	An observational online survey	122 patients newly diagnosed with lung cancer	To identify sociodemographic and clinical characteristic of lung cancer patients, focusing on aging‐related factors as high‐risk factors of influence on impaired decision‐making capacity	1. *Decision‐making preferences*: *MacArthur Competence Tool for Treatment (MacCAT‐T)* 2. *Cognitive impairments*: *Mini‐Mental State* *Examination (MMSE)* 3. *Depression symptoms*: *Patient Health Questionnaire‐9 (PHQ‐9)* ^28^ 4. *Frailty*: *Vulnerable Elders Survey‐13*	The MacArthur Competence Assessment Tool for Treatment (MacCAT‐T) was and instrument that measures: (a) understanding of the disorder and its treatment, (b) appreciation of the disorder and its treatment, (c) reasoning and ability to compare alternatives, and (d) the ability to express a choice	Patients with impaired decision‐making preferences had significant deficits in cognitive and executive functions and frailty. Moreover, they performed worse on the assessment of decision‐making capacity than those without cognitive impairment. No significant association was identified between depression and decision‐making capacity
7. Olling et al. (2019)^29^	A real‐life observation study	29 patients with lung cancer	To explore whether a DAs improved SMD and supported a patient‐centered approach from an observation point of view	1. *Shared decision‐making*: OPTION 12 Scale 2. *Patients preferences*: OPTION 12 Scale	Decision aids were based on the implementation of a DAs template designed to support shared decision‐making in the choices of adjuvant therapy	The DAs implementation improved shared decision‐making on adjuvant treatment in lung cancer. Patients specifically expressed their preferences in being engage in their oncological treatment decisions
8. Søndergaard et al. (2019)^30^	A prospective cohort studies	52 lung cancer patients	To compare an unexposed cohort with an exposed cohort to SDM and an in‐consult DAs with lung cancer patients.	1. *Decisional regret*: *a validated 5‐item Decision Regret Scale (DRS*)^18^, ^27^, ^31^ 2. *Decisional Conflict* *Decision Conflict Scale (DCS)* 20, 21	The International Patient Decision Aid Standards (IPDAS), based on a four‐step approach (i.e., supports choice talk, preference talk, option talk, and decision talk) was implemented	Patients experienced less decisional regret thanks to higher levels of decisional engagement. At the same time, decisional conflict and regret decreased in a high‐volume, fast‐track lung cancer diagnostics organization
9. Wu et al. (2020)^32^	A non‐randomized clinical trial	A cohort of 76 NSCLC	To evaluate an interactive web‐based tool, aiming at allowing patients to explore tailored decisions	1*. Decisional Conflict*: *Decision Conflict Scale (DCS)* ^20^, ^21^ 2. *Decision‐making preferences*: *Decision‐making preferences Questionnaire* 3. *Quality of life*: *Functional Assessment of Cancer Therapy‐Lung (FACT‐L)* 4. *Satisfaction with decision*: 6*‐item questionnaire measuring satisfaction*	The decision tree was based on an interactive calendar‐like timeline that allowed patients to map out their sequence of treatments over time	Tool implementation during consultation was associated with decreased decisional conflict and greater satisfaction with patients' decisions. Half of patients expressed that they and their physician should take equal part in the decision‐making process. The use of DSI improved the Quality of Life of patients, who generally felt supported by their families and clinicians
10. Yılmaz et al. (2019)^28^	A between‐subjects factorial study	305 NSCLC patients	To assess the effects of DAs based on audiovisual and narrative information in older patients compared to younger patients	1. *Perceived cognitive load*: *An adapted 4‐item scale measure* 2. *Satisfaction with information*: *Website Satisfaction Scale* 3. *Patients' knowledge*: *8 multiple‐choice questions* ad hoc 4. *Informational recall*: *15 open questions that based on the* Netherlands Patient Information Recall Questionnaire 5. *Decisional conflict*: *Decisional Conflict Scale (DCS)* ^20^, ^21^	Audiovisual and narrative information of an early‐stage NSCLC treatment decision aid explained surgery and stereotactic ablative radiotherapy to patients	Audiovisual information reduced perceived cognitive load. At the same time, satisfaction with information and patients' knowledge were increased. Particularly, audiovisual information resulted not only in less perceived cognitive load, but also in more patients' satisfaction and attractiveness to the information than textual information. Finally, less decisional conflict and better comprehension and recall information were found

### 2.5 Quality assessment of the studies

The quality of the selected studies was also assessed through the guidelines of the Cochrane risk of bias tool, version 2 RoB 2.^33^ We evaluated the Quality of the selected study following the Cochrane risk of bias tool toll, version 2RoB 2, which is based on quality appraisal and their biases and domains.^23^ The assessment of the methodological quality of each study was conducted by two researchers (AP and LP) independently and all the results are summarized in Table 2. Items were scored as “+” if the criterion was met, “?” if it was uncertain, and “‐” if it was not met. There is a low risk when the risk assessment related to all the domains is scarce.^28^ A discussion with a third author (FT) solved disagreements between raters.

**TABLE 2:** Cochrane risk of bias tool.

	Random sequence generation	Allocation concealment	Blinding of participants and personnel	Blinding of outcome data	Incomplete outcome data	Selective reporting	Other bias
1. Brundage et al. (2001)^17^	‐	‐	‐	‐		‐	‐
2. Fiset et al. (2000)^22^	‐	‐	‐	‐	‐	+	‐
3. Hollen et al. (2012)^23^	‐	‐	‐	‐	‐	‐	+
4. Leighl et al. (2008)^25^	+	‐	‐	‐	‐	‐	‐
5. Ogawa et al. (2018)^27^	‐	‐	‐	‐	‐	‐	?
6. Olling et al. (2019)^29^	‐	‐	‐	‐	‐	‐	‐
7. Myers, et al. (2021)^26^	‐	+	‐	+	‐	‐	‐
8. Søndergaard et al. (2019)^30^	+	‐	‐	‐	‐	+	‐
9. Wu et al. (2020)^32^	‐	‐	‐	‐	‐	‐	‐
10. Yılmaz et al. (2019)^28^	‐	‐	‐	‐	‐	‐	‐

*Note*: “+” = Low risk of bias; “?” = Unclear risk of bias; “‐” = High risk of bias.

### 2.6 Outcome measures

Multiple variables were reviewed in the majority of the studies. Ten reviewed articles are presented in Table 1, which shows primary outcomes in the last column. Two of the authors (ND and ES) performed this results categorization independently; a third reviewer (VS) resolved any disagreement. Thus, outcome variables and their related theoretical constructs can be highlighted. Improvement in patients' decision‐making processes and well‐being in health management can be related to the implementation of specific and tailored tools during medical consultation. This way, intervention effectiveness can be evaluated on tracked data. Studies were quite heterogeneous, but some interesting patterns can be reported. Decision‐making was analyzed by the majority of the reviewed studies (*n* = 9); patient knowledge was the second most analyzed outcome (*n* = 6), followed by anxiety scores (*n* = 2). Finally, six studies reported other improved results.

## 3 RESULTS

### 3.1 Characteristics of interventions

This systematic review includes studies conducted in different countries. To be specific, it includes research in the United States (*n* = 5), Europe (*n* = 4), and Asia (*n* = 1). Considering all studies involved, 20 to 270 participants were included. Four of the studies indicated a sample size of fewer than 50 participants, and five articles involved between 50 and 100 participants. Only one study showed a sample comprising over 100 participants. Furthermore, all the studies included lung cancer patients either exclusively or, at the very least, as one of the experimental groups.

Two articles involved at least one control group; at the same time, eight other studies employed one‐group subject designs or cross‐sectional research. Regarding PDAs, studies confirmed that this category could enhance patient well‐being through various mechanisms. The main outcomes obtained were decreased anxiety (*n* = 2) and improvements in decision‐making, in terms of decision‐making satisfaction (*n* = 2) and quality (*n* = 1), preferences (*n* = 6), regret (*n* = 1), SDM (*n* = 3) and decisional conflict (*n* = 6), patient knowledge about illness characteristics and treatments options (*n* = 4), and other variables (e.g., quality of life, cognitive impairments, frailty, cognitive impairments, information recall, and the tool acceptability [*n* = 5]).

Each study implemented a decision tool with specific characteristics and goals. Particularly, Myers et al.^26^ employed decision tools based on the implementation of a decision support intervention (DSI) designed to support decisions in the choice of a patient's treatment. The authors proposed a new DSI, including care plan cards and companion preference clarification tools. The aim was to promote decisions. In order to do this, the cards were used to answer the most common questions about options of treatments, such as target therapy, immunotherapy, chemotherapy, chemotherapy plus immunotherapy, clinical trial participation, and supportive care. Moreover, cards elicited patients' preferences about their available treatments. In addition, interviews with both patients and physicians were conducted to hear opinions and suggestions on the DSI. Patients described what treatment alternatives they were likely to recommend. Furthermore, they specified how DSI could help them understand their diagnosis, treatment options, and make decisions. Moreover, The DSI prototype was presented to physicians, who were asked to provide feedback regarding its feasibility in clinical care.

Sondergaard and colleagues^30^ developed a specific patient decision aid (PDA) to be used during the diagnostic workup. The PDA initially informed patients that a choice needed to be made, then asked how much information they preferred to receive (i.e., “a minimum of information, a moderate amount of information, and the most information possible”). Then, patients specified their value, choosing between two options: “Rapid clarification is more important to me than avoiding the complications involved in the diagnostic program” or “Avoiding the complications involved in the diagnostic program is more important to me than rapid clarification”. Lastly, we investigated whether patients were ready to make decisions in collaboration with their physicians. Two studies included in this systematic review,^29^, ^32^ developed a PDA template to support lung cancer patients' preferences, but in a web‐based format. In particular, Olling et al.^29^ developed a PDA model to support SDM in the diagnostic workup. It was then tested in accordance with the systematic model proposed by Coulter et al.^34^ Moreover, it explored fears, expectations, and general questions that lung cancer patients had about their lung cancer diagnosis. The web‐based “build‐your‐own‐PDA” software platform made PDAs more accessible for all patients and healthcare professionals.

Similarly, Wu et al.^32^ employed a web‐based PDA tool to elicit patient preferences. It presented an interactive web‐based interface in which patients could enter their illness features, such as clinical, pathological, and radiographic characteristics. In this step, they were assisted by a trained research coordinator. At the same time, patients could explore available combinations and sequences of treatments following the national health guidelines. Additionally, an interactive calendar‐time timeline was created thanks to a decision tree. It was provided in order to map out the patients' sequence of treatments over time. As a consequence, the authors established certain quality care benchmarks. In order to do this, guidelines were followed (e.g., adjuvant chemotherapy, documented smoking cessation counseling, or pathologic mediastinal staging for patients after surgery, and molecular testing). Furthermore, the PDA developed by Hollen et al.^35^ was called “Decision‐KEYS for Balancing Choices” and was inspired by the Janis and Mann' conflict theory of decision‐making (1977),^36^ which predicts decision‐making behaviors for consequential decisions. It included common choices related to lung cancer patient care. For example, changing chemotherapy and type of lung surgery. This specifically pertained to crucial categories related to treatment options, which could be rapidly updated if necessary. Moreover, medical practitioners, including physicians and nurses, meticulously provided patients with in‐depth information about side effects and customized key categories to address individual needs. Brundage et al.^17^ applied a PDA to involve a structured treatments' description and trade‐off exercises. Specifically, the aim was to define patients' values, considering their survival advantage threshold. Seven components were followed in the description of each option of treatment. These were details of the actual regimen of treatment; side effects in the short‐ and long‐term, also considering their frequencies; effects of treatments on emotional states, social relationships, and personal functioning; and available symptoms due to cancer and its related oncological interventions.

Each participant was then asked to express their preferences about treatment options and given the possibility to make an informed treatment decision. Interestingly, three studies included in the present systematic review^22^, ^25^, ^28^ implemented an audio‐recorded booklet as a PDA to explain the harms and benefits of the treatment options. Firstly, Fiset et al.^22^ developed and evaluated a PDA entitled “Making Choices: Treatment of Stage IV Non‐Small Cell Lung Cancer” based on a worksheet and a self‐administered audio‐tape booklet. Moreover, it is focused on the Ottawa Decision Support Framework and, in particular, three of its basic strategies, as follows: (1) sharing information about treatment options and outcomes to increase patients' knowledge and support realistic expectations; (2) providing clinical examples to increase skills in making decisions; and (3) involving a “weight‐scale” exercise to sustain patients in clarifying and communicating their personal values. Patients were guided by an audio‐tape for 35 min through the revision of booklet' information. Then, they filled out a personal worksheet. Specifically, the PDA showed illustrative icons to represent each available treatment option. Three panels were involved in the PDA creation: a development panel; a practitioner panel; and a patient panel. Moreover, the authors adjusted the text involved into the PDA to make it comprehensible to those with less formal education, supported by illustrations and audio‐tape. Aids were used by patients in a self‐placed fashion. Moreover, they used them alone or with the help of family members.

Second, Leighl and colleagues (2008)^25^ designed a booklet of 25 pages as a PDA tool. This was a letter‐sized booklet that showed treatment options, survival information illustrated in a graphic form, and treatments' toxicity. Additional information included a color‐coded calendar to schedule days of treatments. At the same time, it assessed the most likely toxicity for each treatment and a flowchart about last treatment pathways. Lastly, a list of clinical references was provided. Third, Yilmaz and colleagues (2019)^28^ included an audiovisual and narrative PDA tool to increase patients' knowledge and a more accurate understanding of risk to reduce decisional conflict. The authors discussed the beneficial outcomes of narrative and audiovisual information in both old and young lung cancer patients. Lastly, Ogawa et al.^27^ presented a decision tool inspired by the MacArthur Competence Assessment Tool for Treatment (MacCAT‐T),^37^ which explored information needed to decide patients' values and preferences. A semi‐structured interview measured decision skills evaluating four domains: (a) the knowledge of the illness and its available treatments, (b) its related appreciation; (c) reasoning and individuals' ability in comparing available alternatives; and (d) individuals' abilities to share a personal choice.

In particular, four themes emerged, outlined as follows:

#### 3.1.1 Decision‐making

Firstly, nine of the reviewed studies^17^, ^22^, ^26^, ^27^, ^28^, ^32^, ^35^ demonstrated improvements in decision‐making due to the implementation of PDAs; this is considered significant in the published literature, highlighting the relevance of decision‐making, for example, in scenarios involving multidisciplinary teams operating at the institutional level.

Specifically, studies in the present systematic review involved decision‐making regarding patients' quality of life, satisfaction, and preferences, decisional conflict, and SDM after the implementation of PDAs about cancer treatments. In particular, Wu et al.^32^ described the feasibility and efficacy of a web‐based, customizable decision support tool that facilitates direct access to health guidelines. Implementing a decision support tool led to an interactive calendar‐like timeline to map out patients' sequences of treatments over time. In this way, patients could find out their available treatment options, taking into account of their personal preferences, with a good balance between patients' and physicians' expression of preferences in the decision‐making process. Consequently, patients reported uncertain feelings about their preference choice and high satisfaction with their treatment decisions. Additionally, greater reductions in decisional conflict allow patients to increase their access to guidelines at a higher frequency after medical consultation. Similarly, Yilmaz and colleagues^28^ reported improvements in patient satisfaction, expression of treatment preferences, and less decisional conflict. On the contrary, patients preferred health information presented in an audiovisual or textual form. Furthermore, decisional uncertainty scores decreased, indicating enhanced satisfaction with the information received and a heightened perception of decision effectiveness. This improvement can be attributed to better comprehension and recall of knowledge. Third, Fiset et al.^22^ addressed patients' preferences and decisional conflict by implementing a self‐administered and audio‐tape booklet, a PDA tool that improved patients' knowledge about treatment options and outcomes. The authors demonstrated that the booklet was fundamental to decreasing patients' uncertainties about their health choices. Moreover, patients reported that they were likely to use the booklet, confirming its feasibility and their final satisfaction. Fourth, Brundage et al.^17^ demonstrated that PDAs can promote patients' preferences and reduce decisional conflict due to an accurate and structured description of treatment options ‐off exercises, which enhanced the comprehension of pertinent information regarding treatment outcomes and advantage thresholds. In particular, the authors reported that patients who had a clear treatment preference at the beginning of the interview did not change their choice after using a PDA tool. However, PDAs were fundamental to addressing patient uncertainty or the lack of information.

Regarding decisional regret, Søndergaard et al.^30^ described the implementation of the International Patient Decision Aids Standards based on a four‐step approach to SDM. In the study, medical consultations were likely to last a few minutes longer. However, patients significantly promoted SDM and engagement, declining unnecessary treatment or evaluations without a sense of regret. Patient preferences were also assessed by Ogawa and colleagues (2018),^27^ who demonstrated how PDAs can promote an individual's ability to choose cancer treatments based on individual preferences. Similarly, a study carried out by Olling et al.^29^ improvements in patient preferences and SDM. To be specific, PDAs improved shared decision‐making adjuvant treatment about and diagnostic workup in a fast‐track lung cancer pathway. This stands in contrast to situations where physicians inaccurately gauge patients' inclination for active involvement in their healthcare decisions.

Consistent with this, patients affirmed their preference for active engagement in treatment decisions. Myers et al.^26^ illustrated that a novel patient‐friendly PDA can be seamlessly and effectively incorporated into routine care, reducing decisional conflict and increasing SDM. Patients experienced heightened certainty regarding their preferences for specific treatments, thereby minimizing decisional conflict. Lastly, in the study conducted by Hollen et al.,^35^ it was observed that decisional conflict decreased, in association with low levels of uncertainty about the choice of cancer treatments. At the same time, interviews with patients and their caregivers revealed high scores in decision‐making quality attributable to the implementation of PDAs.

#### 3.1.2 Anxiety

Second, two of the reviewed studies^25^, ^33^ demonstrated that PDAs decrease patient anxiety. In particular, Leighl et al.^25^ demonstrated that the presentation of a booklet with treatment options during and after medical consultation could be a helpful tool to reduce patient anxiety. Interestingly, none of the patients had a baseline anxiety level that warranted exclusion by the physician. Patient anxiety decreased slightly after implementation of a PDA due to improved understanding of goals and side effects of cancer treatments in advanced NSCLC. Additionally, authors showed the relevance of maintaining or promoting patient hope, despite prognosis and invasive treatments.

Similarly, in the study by Hollen and colleagues,^23^ patients and their related caregivers who were interviewed after medical consultation reported high anxiety levels at the initial interview; however, anxiety scores decreased after PDA implementation, compared with the average. It may be noted that no significant differences in anxiety comparing patients and their caregivers were shown.

#### 3.1.3 Patient knowledge

Six studies that applied PDAs demonstrated improvements in patient knowledge of disease characteristics and available treatment options, promoting a better balance of their goals of care.

In the study by Leigh and colleagues,^25^ patients with advanced NSCLC showed a clear understanding of the aims and side effects of cancer treatments (e.g., toxicity) after consulting PDAs. Authors expressed that understanding the prognosis, treatment options (involving or not chemotherapy, for example), health outcomes, and supportive social care are essential. Interestingly, there was no evidence about the impact of sociodemographic data (e.g., educational attainment, age, and gender) on patients' knowledge (e.g., gender, educational attainment, English‐speaking background, age, prior chemotherapy, and baseline anxiety). Moreover, Yilmaz and colleagues^28^ demonstrated that providing PDAs such as audiovisual and narrative information enhanced the understanding and application of health‐related information of early‐stage NSCLC treatment. Interestingly, the modality of presentation had no significant effect on patient satisfaction and comprehension. Moreover, authors showed that the interaction between tool modality, narration style, and age significantly affected the perceived cognitive load. In other words, irrespective of age, authors demonstrated that audiovisual information about treatment risks and benefits promoted the patients' awareness in comparison to textual information. It is possible that audiovisual information supported patients' comprehension and recall in a more efficacious way. Moreover, the authors hypothesized that information exceeded the capacity of the working memory in younger patients. Thus, similar outcomes regarding audiovisual information can be a positive finding for older and younger patients and can be interpreted as a positive result for health management's objectives. Accordingly, Brundage et al.^17^ showed that clarifying the information imparted to patients is fundamental in promoting their treatment decisions. Lastly, two other studies^22^, ^26^ reported that their PDA prototype had a beneficial effect on the patient perception of cancer treatments, thanks to a better awareness of treatment options.

#### 3.1.4 Other variables

Finally, six of the reviewed studies^22^, ^25^, ^27^, ^28^, ^32^ also showed improvements in other variables, namely: Quality of Life, cognitive impairment, frailty, cognitive impairments, information recall, and the tool acceptability. Referring to Quality of Life, Wu et al.^32^ showed that PDAs increased patients' perception of support from their families and friends, leading to a moderate association between lung cancer diagnosis and their functional well‐being daily. Moreover, Fiset et al.^22^ reported the feasibility and acceptability of PDA tools, feeling patients were comfortable in using them. Patients explicitly expressed that they were likely to implement this tool. Accordingly, Leighl et al.^25^ and Hollen and colleagues^23^ results evidenced the tool's feasibility for lung cancer patients during medical consultation on oncological treatments. Additionally, Ogawa et al.^27^ showed fewer cognitive impairments and deficits in executive functions during the use of PDAs. At the same time, authors demonstrated improvement in patient perception of frailty, whereas no significant findings were found regarding depression scores. Lastly, as previously reported, Yilmaz and colleagues^28^ demonstrated that PDAs can reduce cognitive load and increase the effectiveness of health information recall.

## 4 DISCUSSION

Consistent with the prior literature, multiple heterogeneous DA tools have been developed and tested during patient‐and‐oncologist treatment decisions. No one specific tool has been used in the field of NSCLC exclusively, but multiple PDAs have been implemented in NSCLC. Moreover, the application of PDA tools has demonstrated generally encouraging outcomes considering the promotion of emotional well‐being, SDM, and other variables of interest related to health measures. SDM has been linked to reduced decisional regret and decreased anxiety regarding treatment choices, improved health outcomes, and increased patient satisfaction. However, like any innovation, implementing SDM requires healthcare professionals to adapt to overcome barriers such as time constraints, perceived limitations in specific cases, and cultural shifts (Ankolekar et al., 2018).^38^

The present systematic review reported a strong association between decisional conflict and patients' uncertainties regarding option treatment options.^17^, ^22^, ^26^, ^28^, ^32^, ^33^, ^39^ This aligns with the study by Nugent et al.,^40^ the association between low decisional conflict and uncertainties could be explained by high‐quality patient‐and‐doctor communication, which also promotes patients' self‐efficacy. In this way, trust in physicians can impact decisional conflict, influencing higher treatment adherence.^18^, ^41^ However, we did not find relevant and significant associations between depression and decision‐making in one of the included studies.^23^ Similarly, a study by Cooley and colleagues^3^ did not find an association between improvements in patient symptom assessment and management and health outcomes regarding emotional well‐being and quality of life. Patients' knowledge, such as a specific focus of some of the reviewed articles, appears heavily relevant for lung cancer patients, in accordance with the literature.^42^ For example, the authors demonstrated the relevance of training‐related knowledge through educational animation to promote exercise compliance. Moreover, Leighl et al.^25^ explained that discussing setting realistic expectations about outcomes after oncological treatments and, in general, life expectancy is fundamental to increasing patients' knowledge. In other words, promoting or sustaining hope in lung cancer patients effectively can result in better understanding after PDA. In keeping with this, Hauber et al.^43^ identified the importance of hope. In particular, they considered hope a relevant value among patients who have a chronic illness, underscoring their priorities when choosing treatments.

In addition, there is a strong connection between improvements in social support, emotional support (which is closely related), and an increased quality of life in lung cancer patients, especially regarding anxiety. Hofman et al.^44^ that social support is essential to increase quality of life in lung cancer patients, sustaining the possibility of minimizing illness symptoms, which to many is more important than length of life. Social support reduces distress by enhancing coping strategies. Accordingly, positive social support can decrease patients' anxiety, thanks to the perception of being emotionally supported by others.^19^, ^20^, ^44^ In the present review, caregivers can support patients in reviewing the DA tools by dedicating appropriate time to reflecting on its contents at home.^25^ Keeping with the published literature, the implementation of a booklet with the support of peers showed significant improvements in distress management.^24^

Lastly, the present study highlights the relevance of understanding and appreciating information. Results suggest that general cognitive function abilities predict decision‐making capacity,^27^ as supported by the biomedical literature.^42^, ^45^ Therefore, patients who show difficulties in understanding information could benefit from enhanced support, such as receiving information through multiple methods (e.g., hearing and seeing). Moreover, patients could be provided with summaries of information and enough time to paraphrase what was explained, reviewing information again with the physician.^42^, ^45^ Accordingly, Kutzleben et al.^46^ demonstrated that involving caregivers was extremely helpful. Similarly, taking enough time is fundamental. At the same time, investigators have suggested that it is important for patients to be able to contact their healthcare practitioners regularly between treatment cycles to improve their understanding of information. In this regard, it would be helpful to involve a multidisciplinary team that could provide all relevant information regarding lung cancer and its related treatment options in order to guarantee fully aware decisions.^37^ Clinicians may need to acquire new skills in facilitating decision‐making discussions that recognize the expanded role of the patient in the SDM process. Previous research suggests two primary skill areas: relational competencies (establishing effective interactions between clinicians and patients) and risk communication competencies (helping patients comprehend treatment options and associated risks). Relational competencies, such as proficient communication, can be honed through training programs, e‐learning modules, or simulated patient encounters to acquaint physicians with the fundamental aspects of SDM. Risk communication competencies entail developing effective methods to articulate treatment‐related probabilities within decision‐making discussions beyond what is provided in PDA (Ankolekar et al., 2018).^38^ According to this study, Joseph‐Williams et al. (2017)^47^ also emphasize that a critical aspect of implementing SDM is to improve understanding of its components. Clinical teams need assistance in evaluating their current practices, fostering a collective understanding of the differences between their current approach and SDM, and determining their preferred approach to decision‐making with patients. It was found that interactive skills training workshops, based on an SDM model, helped establish coherence, improve skills, and promote positive attitudes.

### 4.1 Study limitations

This systematic review has multiple limitations. Although three databases are generally regarded as sufficient for a systematic review,^48^ more research sources on similar issues could be helpful for a more comprehensive approach. Secondarily, opting for a relatively recent time range could enhance the focus on more representative and current PDA tools. Furthermore, future review efforts might consider employing alternative search strategies or adopting a different perspective on the present contribution to explore additional lung cancer‐specific outcomes related to treatment decisions and the implementation of PDAs during lung cancer consultations. Accordingly, future research could include qualitative studies aimed at furnishing a broader overview of this topic of interest. In accordance with this, a research method to capture the patient perspectives is fundamental. Future studies could lead to guidelines being drawn up for new PDAs, improving the effectiveness of future tools. Thus, a meta‐analysis could be conducted to better assess differences in the effectiveness of the PDA tools. Moreover, the present systematic review does not include only randomized control trials: it would be indeed interesting to select only articles that follow a “gold standard” methodology to better assess clinical outcomes. Importantly, only one study analyzed in this review included immunotherapy and other novel therapeutic approaches such as target therapies in the PDA. Since the therapeutic landscape in lung cancer has dramatically changed over the past decade in terms of efficacy and tolerability, an urgent need to create a new tool able to help patients with novel treatment choices is emerging. Projects aiming to fill this scientific gap are ongoing (NCT05537922).

## 5 CONCLUSION

In conclusion, this systematic review highlights the utility of various PDA tools as valuable resources in lung cancer treatment consultations. The outcomes indicate a positive impact resulting from the implementation of PDA tools, affirming their role in enhancing the overall quality of patient–doctor consultations regarding cancer treatments. Notably, the review demonstrates the effectiveness of different tools, such as decisional trees, booklets, and audiovisual information, in addressing specific health issues. Additionally, considering the nature of chronic conditions, effective patient–doctor communication, treatment adherence, and social support are crucial elements that should be integrated into PDA approaches.

### 5.1 Clinical implications

Involving patients in the decision‐making process may prove helpful in achieving better outcomes in their healthcare management. This starts with assessing their preferences and motivations for engagement in the cancer treatment pathway.^49^ Such involvement could be relevant in promoting psychological well‐being among lung cancer patients.

## AUTHOR CONTRIBUTIONS

**Valeria Sebri:** Conceptualization (equal); data curation (equal); methodology (equal); project administration (equal). **Chiara Marzorati:** Conceptualization (equal). **Patrizia Dorangricchia:** Formal analysis (equal). **Dario Monzani:** Conceptualization (equal); resources (equal). **Roberto Grasso:** Data curation (equal). **Arsela Prelaj:** Resources (equal). **Leonardo Provenzano:** Conceptualization (equal). **Laura Mazzeo:** Visualization (equal). **Andra Diana Dumitrascu:** Methodology (equal). **Jana Sonnek:** Project administration (equal). **Marlen Szewczyk:** Validation (equal). **Iris Watermann:** Investigation (equal). **Francesco Trovò:** Writing – original draft (equal). **Nina Dollis:** Supervision (equal). **Evangelos Sarris:** Formal analysis (equal). **Marina Chiara Garassino:** Writing – original draft (equal). **Christine M. Bestvina:** Methodology (equal). **Alessandra Pedrocchi:** Methodology (equal). **Emilia Ambrosini:** Supervision (equal). **Sokol Kosta:** Resources (equal). **Enriqueta Felip:** Validation (equal). **Mireia Soleda:** Writing – original draft (equal). **Aina Arbusà Roca:** Conceptualization (equal). **Jose Rodríguez‐Morató:** Methodology (equal). **Alessandro Nuara:** Writing – original draft (equal). **Yonah Lourie:** Writing – original draft (equal). **Melissa Fernandez‐Pinto:** Methodology (equal). **Alfonso Aguaron:** Project administration (equal). **Gabriella Pravettoni:** Supervision (equal).

## CONFLICT OF INTEREST STATEMENT

There is no conflict of interest.

## FUNDING INFORMATION

The I3LUNG project has received funding from the European Union's Horizon 2020 caLL topic “HORIZON‐HLTH‐2021‐CARE‐05‐02—Data‐driven decision‐support tools for better health care delivery and policy‐making with a focus on cancer” under grant agreement No 101057695.

## CLINICAL TRIAL REGISTRATION

Based on the specific nature of this study, no original data was collected, and no clinical trials were performed.

## ETHICS STATEMENT

N/A since the present manuscript is a systematic review. No human subjects were involved.

## PATIENT CONSENT STATEMENT

N/A since no human subjects were involved.

## DATA CITATION

All studies involved in the systematic review have been cited.

## Data Availability

N/A since, based on the specific nature of this study, no original data were collected.
